# Cyclin D2 – a potential biomarker in uterine corpus endometrial carcinoma through methylation chip and bioinformatic analysis

**DOI:** 10.3389/fonc.2025.1569782

**Published:** 2025-07-03

**Authors:** Lizhen Wang, Wu Qu, Zhifu Yang

**Affiliations:** ^1^ Department of Pathology, School of Basic Medicine and Forensic Medicine, Baotou Medical College, Baotou, Inner Mongolia, China; ^2^ Department of Pathology, The First Affiliated Hospital of Baotou Medical College, Baotou, Inner Mongolia, China; ^3^ Department of Neurology, The First Affiliated Hospital of Baotou Medical College, Baotou, Inner Mongolia, China

**Keywords:** uterine corpus endometrial carcinoma, cyclin D2, differentially methylated positions, biomarker, methylation chip

## Abstract

**Background:**

The incidence and mortality of uterine corpus endometrial carcinoma (UCEC) is increasing. Despite advances in diagnosis and treatment, the fundamental molecular mechanisms remain unclear to some extent. In this study, the role and clinical significance of Cyclin D2 (CCND2) in UCEC is discussed and explored.

**Methods:**

The Infinium Methylation EPIC v2.0 BeadChip (935K chip) was utilized to analyze genomic DNA samples from four UCEC patients and four matched controls. Differentially methylated positions (DMPs) were identified, leading to the selection of the CCND2 gene as a candidate gene. Immunohistochemistry (IHC) was employed to validate the effects of CCND2 on UCEC. An analysis was conducted using the UALCAN and GSCA databases to compare the expression and methylation levels of the CCND2 gene promoter region between UCEC and adjacent normal tissues, as well as to explore the relationship between CCND2 expression and the methylation level of its gene promoter region. Subsequently, Cox regression and ROC analysis were performed with R software.

**Results:**

Through 935K chip detection, a total of 87,182 DMPs were identified in the whole genome of two groups. CCND2 was selected for further functional analysis. IHC results revealed that the positive expression of CCND2 in UCEC was significantly lower than in normal endometrial tissue (P < 0.05). TCGA datasets were analyzed to explore differential patterns involving mRNA and DNA methylation features associated with CCND2. The findings demonstrated a significant increase in the methylation level of CCND2 (P < 0.001), and a significant reduction in its mRNA expression (P < 0.001). Furthermore, the methylation level of the CCND2 gene promoter region exhibited a negative correlation with its mRNA expression (Cor. = -0.18, FDR = 0.018). Results from ROC analysis and survival analysis indicated that CCND2 expression was a prognostic indicator for UCEC (AUC = 0.956), with better survival in the high expression group (P = 0.0466).

**Conclusion:**

The study shows that UCEC has significantly abnormal DNA methylation patterns and expression profiles. Hypermethylation of the CCND2 promoter may reduce CCND2 expression and participate in tumor occurrence and development in UCEC. Hence, CCND2 shows promise as a potential biomarker for diagnosing and prognosticating UCEC.

## 1 Introduction

Uterine corpus endometrial carcinoma (UCEC) is a prevalent malignant tumor of the female reproductive system, with its global incidence increasing annually. According to statistics from the American Cancer Society in 2024, 66,570 women were diagnosed with UCEC in 2021. This number is projected to reach 67,880 in 2024, with 4,360 related deaths (1). The development of this disease is closely associated with various risk factors, including prolonged estrogen exposure, metabolic abnormalities (such as obesity and diabetes), and nulliparity. Postmenopausal women, especially those with a family history or Lynch syndrome, are at significantly increased risk (2). Despite advances in understanding the molecular mechanisms of UCEC in recent years, there has been no significant decrease in mortality from advanced UCEC. This lack of improvement may be attributed to the diverse genetic and epigenetic backgrounds of UCEC patients with the same histological type (3). Currently, there is a lack of effective early diagnostic and prognostic markers to determine which patients will benefit most from aggressive treatment. Therefore, the search for biomarkers that can accurately predict the onset and progression of UCEC has become the focus of UCEC prevention and treatment research.

The mechanism of UCEC is intricate, involving a myriad of molecules and cellular signaling pathways (4). Among them, cyclin D2 (CCND2) is an important cell cycle regulatory protein, mainly through the binding of cyclin dependent kinases (CDKs), to supervise the transition of cells from G1 phase to S phase. This pivotal process significantly influences cell proliferation, thereby contributing to tumorigenesis. Studies have shown that abnormal expression of CCND2 is closely related to the occurrence, development and prognosis of various tumors (5). Nonetheless, the expression pattern of CCND2 in UCEC, its correlation with methylation levels, and its impact on prognosis remain unexplored. In this study, gene chip technology, immunohistochemistry (IHC) and bioinformatics were used to investigate the relationship between the methylation level, mRNA expression, and protein expression of the CCND2 gene and UCEC. This approach provides a new perspective for understanding the mechanisms underlying UCEC development and provides valuable insights for advancing early diagnosis and personalized treatment strategies. In-depth study of differential methylation genes in UCEC will not only contribute to our understanding of its pathogenesis but also contribute to the progress of its clinical application.

## 2 Materials and methods

### 2.1 General information

According to the diagnostic criteria of UCEC (6), paraffin-embedded endometrial tissue blocks surgically removed between January 2022 and December 2023 were carefully selected and stored in the Department of Pathology, the First Affiliated Hospital of Baotou Medical College. The cohort included 20 UCEC tissues and 15 normal endometrial tissues, and all participants were between 44 and 75 years of age. This study has been approved by the Ethics Review Committee of Baotou Medical College (Approval number: Baoyi Ethics Human 2021 No. 008), all individuals give informed consent by signing the informed consent form.

### 2.2 Infinium methylation EPIC v2.0 BeadChip (935K chip) assay

Carefully selected paraffin-embedded tissue samples were precisely categorized into the UCEC group and the normal endometrial control group, with essential clinical information meticulously recorded. Subsequently, four samples from each group were precisely sliced, 5 - 10µm thick, for genomic DNA extraction and subsequent on-chip analysis.

DNA extraction was conducted using the QIAamp DNA FFPE Tissue Kit (Catalog no: 56404) following the manufacturer’s protocols. The genomic DNA was then quantified with a spectrophotometer, followed by 1.25% agarose gel electrophoresis. The main band of the sample was more than 10kb and no obvious degradation, and the total amount was more than 3μg, which was suitable for the subsequent methylation chip experiment.

The genomic DNA underwent bisulfite conversion utilizing the Zymo EZ DNA Methylation Kit. Subsequently, the DNA methylation levels of the subjects’ genomic DNA were assessed using the Infinium Methylation EPIC v2.0 BeadChip (935K chip). This advanced chip technology enables the evaluation of methylation status at approximately 950,000 CpG sites across the human genome. The comprehensive detection process, encompassing DNA amplification, fragmentation, precipitation, resuspension, hybridization with the chip, chip washing, single-base extension, staining, chip scanning, and data extraction, was expertly conducted by Beijing Bomiao Biotechnology Co., Ltd.

### 2.3 Candidate gene screening

The chip scan results identified 42 highly methylated loci using thresholds P < 0.0005 and |Δβ| > 0.2 to identify differentially methylated positions (DMPs). Then retrieved from the TCGA database (https://portal.gdc.cancer.gov) and organizational TCGA - UCEC project STAR RNAseq data in the pipeline. Survival regression analysis was carried out by survival package, and the genes related to UCEC prognosis were mainly screened. The significance level was P < 0.05, a hazard ratio (HR) greater than 0.6 and the gene type was protein - coding. A total of 2,457 genes were identified. The DESeq2 software package was used to conduct difference analysis on the original count matrix of RNAseq data, and the differential expression genes in EC were screened. The strict significance threshold was P < 1e-7, an absolute log2 fold change greater than 1.5 and the gene type was designated as protein - coding. Finally, 2,700 genes were identified. The intersection of these datasets revealed two genes, CCND2 and BST1, for further analysis. Subsequently, the function of CCND2 as a major candidate gene was analyzed.

### 2.4 Immunohistochemical detection of CCND2 protein expression

Immunohistochemical detection was executed on the collected 20 cases of UCEC and 15 cases of normal endometrial tissue. The tissues were fixed in 10% formaldehyde solution, routinely dehydrated, cleaned, paraffin embedded, and then continuously sliced to a thickness of 4μm. Subsequent steps included antigen retrieval, incubation with 3% hydrogen peroxide, and serum blocking. A primary antibody (CCND2 rabbit polyclonal antibody purchased from Beyotime: AF6630) was applied sequentially, followed by a secondary antibody, DAB chromogenic reaction, and neutral resin mounting. Optical microscope (Olympusbx53, Tokyo, Japan) was used for observation. To ensure accuracy, PBS was used as a negative control instead of a primary antibody, and the experiment was repeated a minimum of three times.

The degree of immunoreactivity for CCND2 expression was evaluated semiquantitatively on the basis of staining intensity and the proportion of positive tumor cells. Positive cell counts were categorized based on the percentage of stained cells: < 10% positive cells = 0, 10% - 25% = 1, 26% - 50% = 2, 51% - 75% = 3, > 75% = 4. Staining intensity was graded as follows: no staining = 0, light yellow = 1, brown = 2, dark brown = 3. The multiplication of these two parameters determined the expression level as negative (0 points), weak positive (1–4 points), moderate positive (5–8 points), and strong positive (9–12 points). In this study, low expression was identified as negative or weakly positive, and high expression was identified as moderate or strong positive. To reduce scoring bias, the two researchers used blind methods to independently evaluate all parts.

### 2.5 Bioinformatics analysis

The UALCAN database (https://ualcan.path.uab.edu/) was utilized to investigate the methylation and mRNA expression of the CCND2 gene in UCEC and normal endometrial tissues, as well as to examine their association with clinical pathological features (7). The GSCA database (http://bioinfo.life.hust.edu.cn/GSCA/#/) facilitated the online analysis of the correlation between CCND2 gene methylation and mRNA expression. Furthermore, utilizing RNA sequencing data from the TCGA database, the relationship between CCND2 mRNA expression and the expression of methylation regulatory factors was assessed, alongside diagnostic efficacy and survival analysis.

### 2.6 Statistical analysis

An analysis of DMP was conducted using the limma package, which utilizes empirical Bayes statistics to calculate p - values (P.Value). Multiple testing correction was performed using the Benjamini & Hochberg method (adj.P.Val). The thresholds were set at P < 0.05 and |Δβ| > 0.02, with |Δβ| > 0.2 considered indicative of significant differences. Fisher’s exact test was employed to evaluate the protein expression level of CCND2 in UCEC. RNA sequencing data from databases were analyzed using R software, incorporating correlation analysis, Receiver Operating Characteristic (ROC) analysis, and Cox regression. Throughout this study, statistical significance was defined as a p - value less than 0.05.

## 3 Results

### 3.1 Differentially methylated positions analysis

**Figure 1:**
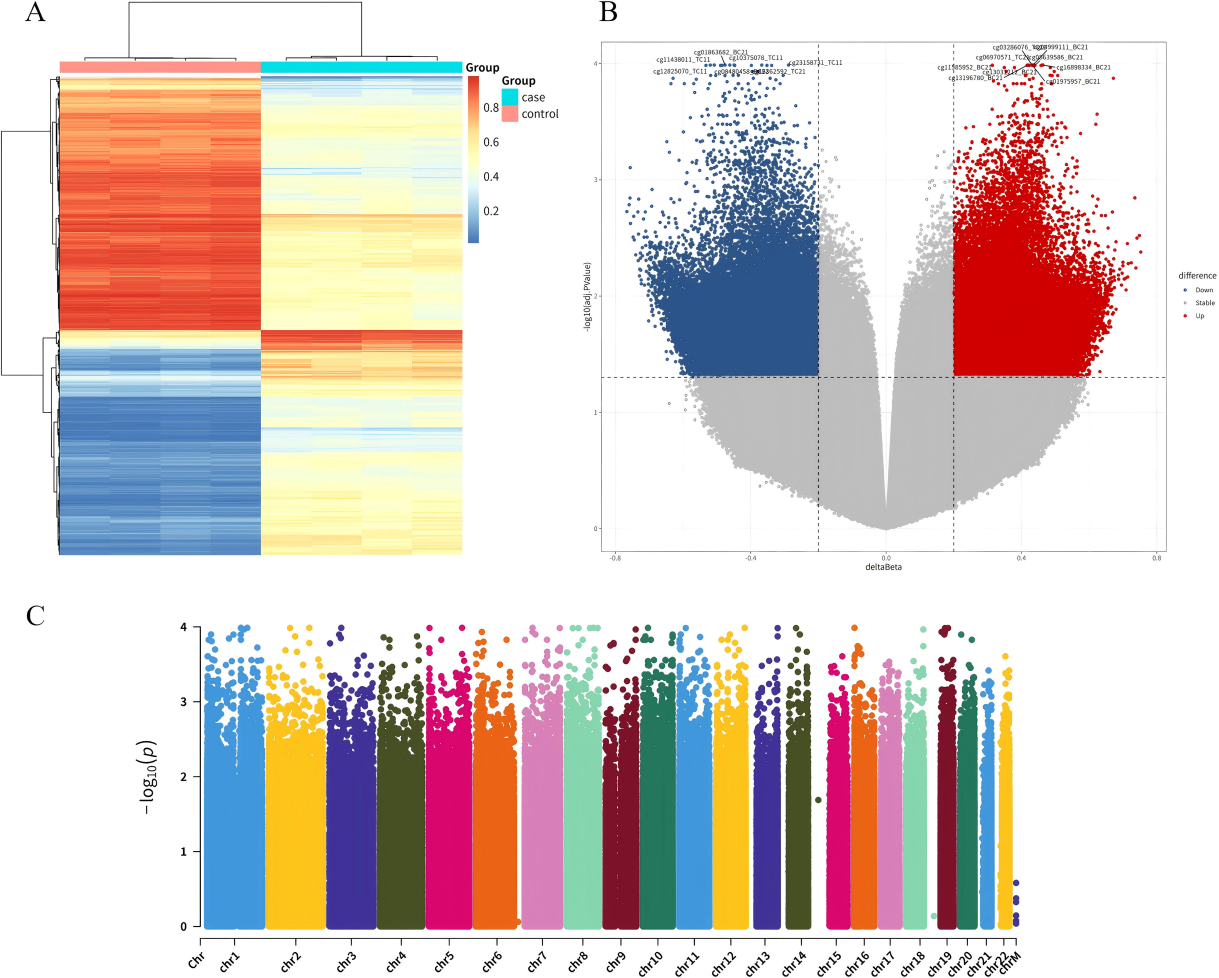
Clustering analysis of DMPs. **(A)** Heatmap clustering of differential loci between UCEC patients and normal controls: The clustering diagram visually reflects the similarity between samples. Each column in the figure represents a sample; each row represents the methylation level of a CpG in different samples, with red indicating relatively high levels and blue indicating relatively low levels; the legend in the upper right corner shows the correspondence between numbers and colors. **(B)** Volcano plot of differential methylation loci between UCEC patients and normal controls: In the volcano plot, the x-axis represents the Δβ values of differential methylation loci, and the y-axis represents the negative logarithm of the adjusted P.Value. **(C)** Manhattan plot across the entire gene range: Comparative analysis of all CpG loci.

935K chip was used to detect high-throughput methylation, and UCEC group was compared with normal control group. After adjusting for P < 0.05 and |Δβ| > 0.02 as thresholds for significant difference sites, 87,182 DMPs were identified, including 41,226 hypermethylated sites and 45,956 hypomethylation sites (**Figures 1A–C**). These locations span the CpG islands, promoter regions, coding regions, and open chromatin regions of all chromosomes. Specifically, 22,483 DMPs were located in promoter regions (Tss1500, Tss200, 3’UTR, 5’UTR, exon 1), of which 13,444 sites showed hypermethylation and 9,039 sites showed hypomethylation. Forty-two hypermethylated gene locations were identified by applying thresholds P < 0.0005 and |Δβ| > 0.2 to DMP.

### 3.2 Functional annotation enrichment analysis of differentially methylated positions

**Figure 2:**
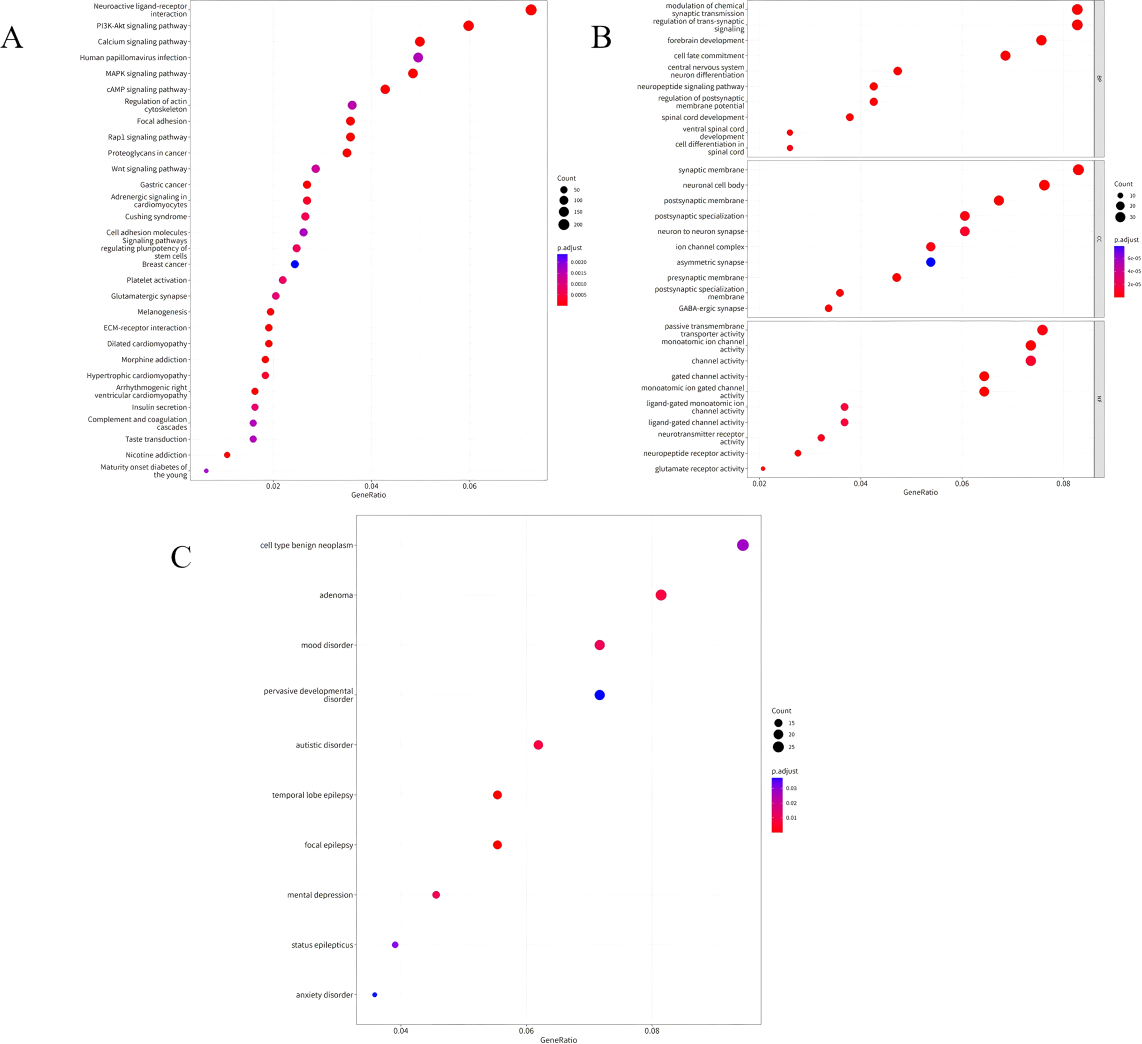
**(A)** Bubble chart illustrating the KEGG enrichment analysis of DMPs in UCEC patients compared to normal controls. **(B)** Bubble chart depicting the GO functional enrichment analysis of DMPs in UCEC patients compared to normal controls. **(C)** Bubble chart showing the disease enrichment analysis of DMPs in UCEC patients compared to normal controls.

The KEGG pathway analysis results of DMPs between UCEC patients and normal controls showed that the DMPs were mainly involved in the PI3K-Akt signaling pathway, calcium signaling pathway, MAPK signaling pathway, and cAMP signaling pathway, as shown in **Figure 2A**. The GO functional enrichment analysis results indicated that the DMPs were mainly concentrated in the modulation of chemical synaptic transmission and the regulation of trans-synaptic signaling in biological processes (BP). In cellular components (CC), they were mainly enriched in the synaptic membrane and ion channel complexes. In molecular functions (MF), they were primarily enriched in channel activity, as shown in **Figure 2B**. **Figure 2C** presented the results of disease enrichment, indicating that these differential positions were closely related to tumorigenesis.

### 3.3 Screen of candidate genes

**Figure 3:**
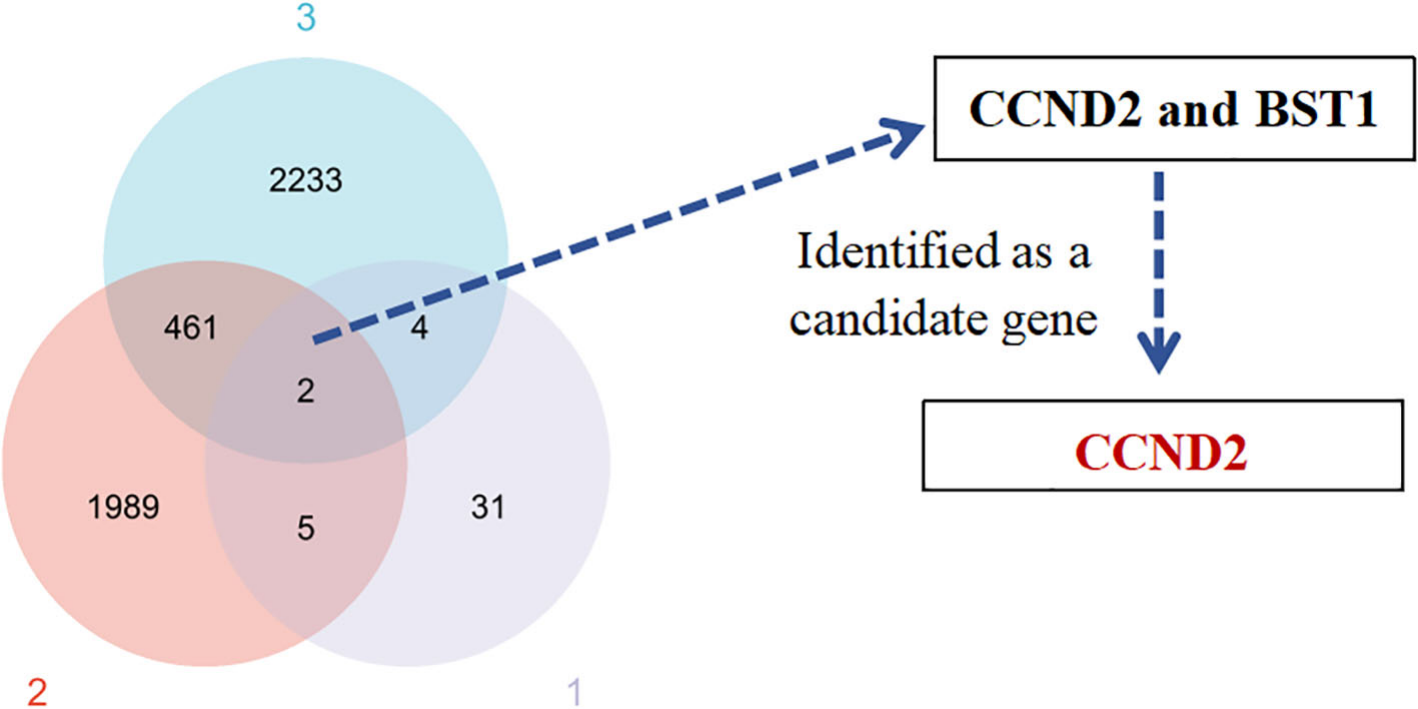
Venn diagram of the identified gene candidates. (1) Differentially methylated genes. (2) Prognostic genes related to UCEC. (3) Differentially expressed genes in UCEC.

The differences of DMPs, prognostic gene and differentially expressed gene between UCEC patients and normal control group were analyzed. The crossover of three datasets (42 highly methylated genes, 2,457 prognostic genes associated with UCEC, and 2,700 genes differentially expressed in UCEC) revealed two common genes: CCND2 and BST1 (**Figure 3**). The CCND2 gene, located on chromosome 12, played a crucial role in the PI3K-Akt signaling pathway and was closely associated with the development of cancer. Based on the results of differential methylation site KEGG signaling pathway and disease enrichment analysis, CCND2 was identified as a candidate gene for further evaluation. This gene is associated with the development and prognosis of UCEC. In the chip data analysis, methylation levels of 7 CpG sites of CCND2 gene were increased, as shown in **Table 1**.

**Table 1:** Differentially methylated sites of the CCND2 gene.

CpG	adj.P.Val	Δβ	CHR	MAPINFO	Gene	cgi
cg00888007_BC21	0.0003	0.26	chr12	4273018	CCND2	Island
cg17296482_TC21	0.0005	0.38	chr12	4273022	CCND2	Island
cg13404421_BC11	0.0005	0.29	chr12	4273015	CCND2	Island
cg15249639_BC11	0.0008	0.26	chr12	4273008	CCND2	Island
cg24626079_TC11	0.01	0.27	chr12	4273005	CCND2	Island
cg18566594_TC21	0.03	0.26	chr12	4272269	CCND2	Island
cg21462428_BC21	0.04	0.31	chr12	4272282	CCND2	Island

CpG, CpG site number; adj.P.Val, p-value adjusted by Benjamini Hochberg; Δβ, difference between the mean methylation level of the UCEC group and the mean methylation level of the control group; CHR, chromosome number where the CpG is located; MAPINFO, physical location; cgi, position of CpG relative to the CpG island.

### 3.4 Expression of CCND2 protein in UCEC and normal endometrial tissue

**Figure 4:**
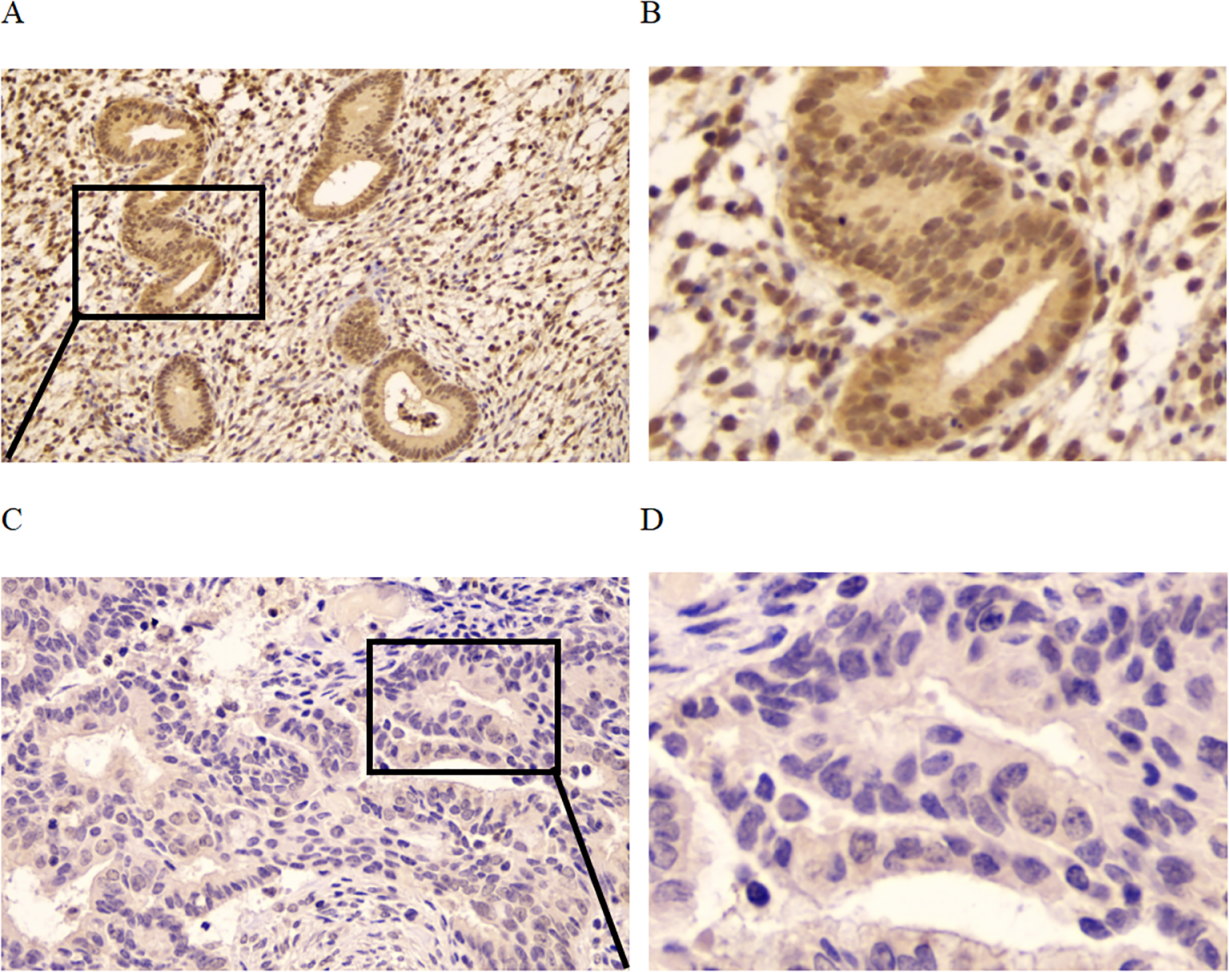
Immunohistochemistry to detect CCND2 expression. **(A)** Normal endometrium was strong positive to CCND2 staining. **(B)** Enlarged area of a normal tissue. **(C)** UCEC tissue was negative to CCND2 staining. **(D)** Enlarged area of a tumor tissue.

The localization and expression level of CCND2 in human UCEC tissues were wished to be determined in the study. Immunohistochemical analysis with an antibody against CCND2 was conducted on 20 human formaldehyde-fixed UCEC tissue samples and 15 normal endometrium tissues. Moderate or strong positive nuclear and cytoplasm staining was detected in glandular epithelial cells in 93.3% of the normal endometrium tissue samples out of 15 cases of normal endometrium tissues, 14 were moderate or strong positive for CCND2 (**Figures 4A, B**). There was only 12 cases with moderate or strong positive CCND2 expression in 20 UCEC cases (**Figures 4C, D**). CCND2 expression demonstrated significant difference between UCEC tissue samples and normal endometrium tissues (93.3% vs. 60%, P = 0.048) (**Table 2**). These results indicate that UCEC tissues exhibit lower levels of CCND2 expression, which is found present in the nuclei or cytoplasm of cancer cells.

**Table 2:** Immunohistochemistry analysis of CCND2.

Characteristics	Total number of cases	Moderate or strong positive	Weak or negative
UCEC tissue	20	12(60%)	8(40%)
Normal endometrium	15	14(93.3%)	1(6.7%)
P-value	0.048*		

* Fischer’s exact test.

### 3.5 Relationship between promoter methylation level and gene expression of CCND2 in UCEC

**Figure 5:**
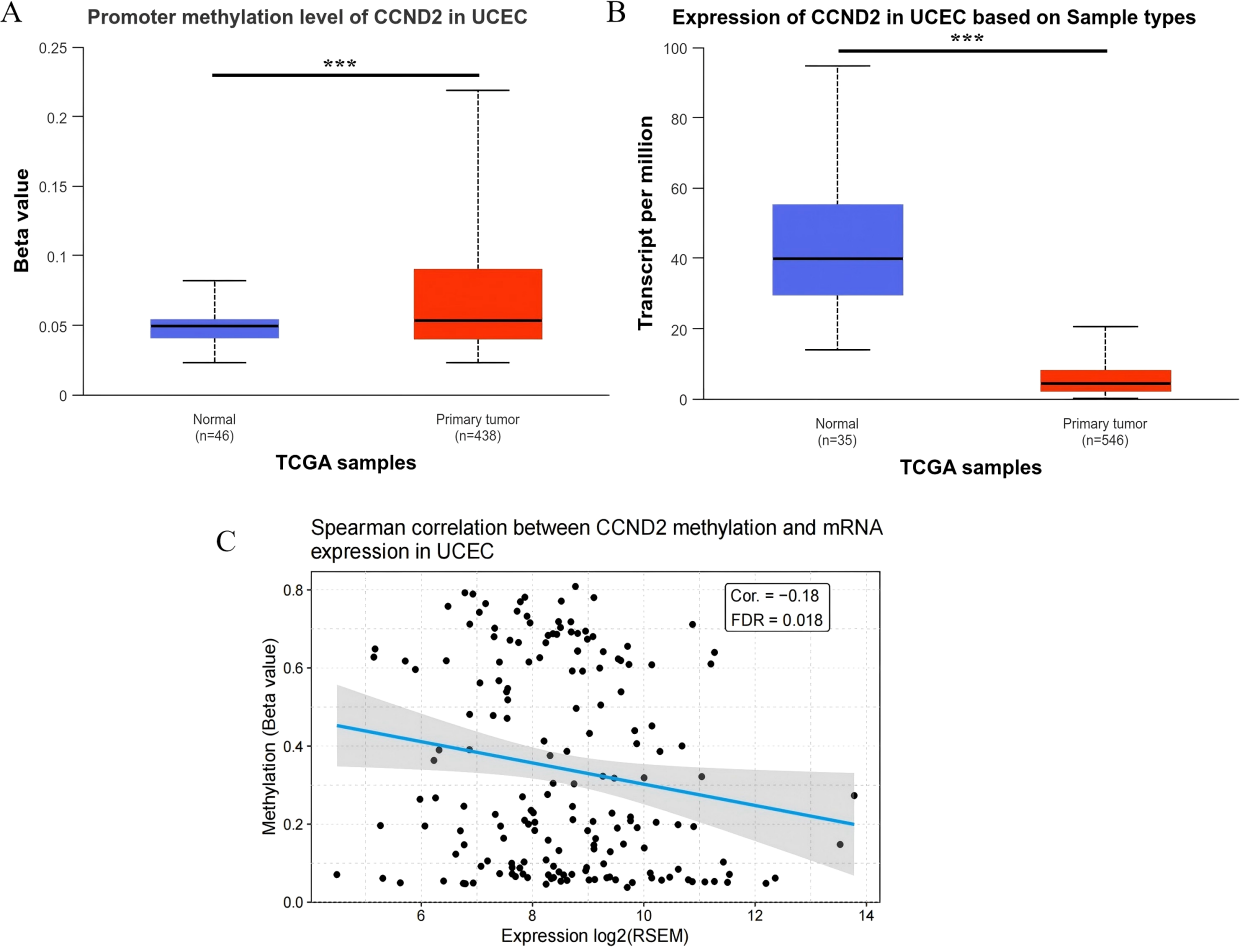
**(A)** Promoter methylation level of CCND2 in normal tissues and tumor tissues (***P < 0.001). **(B)** Expression level of CCND2 in normal tissues and tumor tissues (***P < 0.001). **(C)** Spearman correlation between CCND2 methylation and mRNA expression in UCEC.

TCGA datasets were analyzed using UALCAN to explore differential patterns involving mRNA and DNA methylation features associated with CCND2. The samples without clinical data were excluded, resulting in a final methylation dataset of 438 tumor tissues and 46 normal tissues. The analysis indicated a significant increase in the methylation level of CCND2 in UCEC compared to normal controls (P < 0.001), consistent with the chip results (**Figure 5A**). The database collected CCND2 mRNA data from 546 tumor tissues and 35 normal tissues. The CCND2 mRNA expression in UCEC than in normal tissues was significantly reduced (P < 0.001, **Figure 5B**). The CCND2 mRNA expression in various tumors and their corresponding normal tissues was also examined. The results indicated that the expression levels were variable (**Supplementary Figure S1**). Furthermore, an online analysis revealed that the methylation level of the CCND2 gene promoter region exhibited a negative correlation with its mRNA expression using the GSCA database (Cor. = -0.18, FDR = 0.018), as shown in **Figure 5C**. These results indicate that hypermethylation of the CCND2 promoter may reduce CCND2 expression in UCEC.

### 3.6 Relationship between the promoter methylation levels of CCND2 and clinicopathological features in UCEC

**Figure 6:**
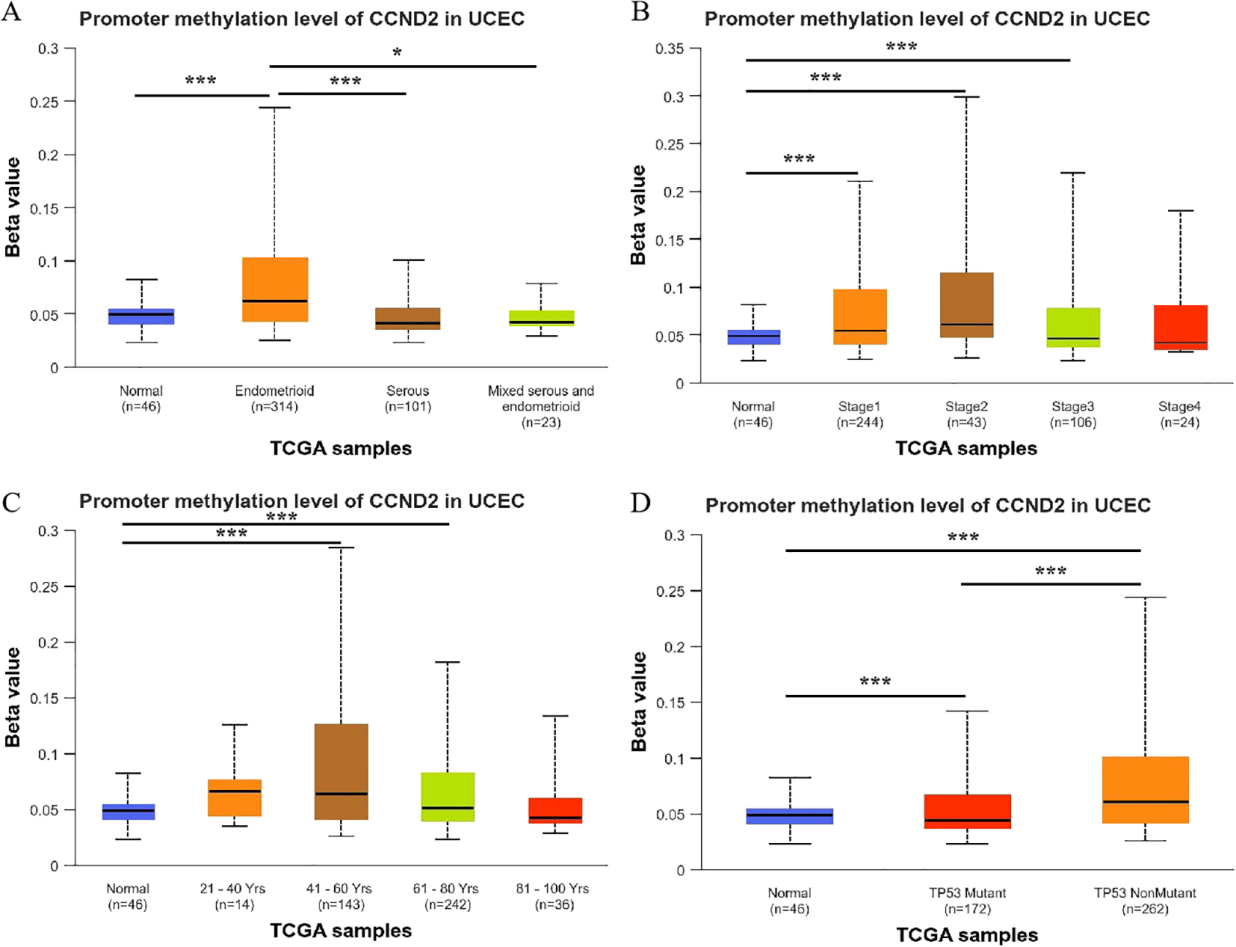
Relationship between the promoter methylation levels of CCND2 and clinicopathological features in UCEC (*p < 0.05, ***P < 0.001).

A detailed analysis of CCND2 gene promoter methylation levels showed a strong correlation with the histological subtypes of UCEC. Notably, endometrioid carcinoma showed significantly higher methylation levels compared to normal tissue, serous carcinoma, and mixed carcinoma (P < 0.001, P < 0.001, P < 0.05) (**Figure 6A**). In clinical stages 1, 2, and 3 of UCEC, CCND2 gene promoter methylation levels were elevated compared to normal tissue, showing a statistically significant difference, P < 0.001 (**Figure 6B**). The methylation levels of CCND2 gene promoter were significantly higher in UCEC patients aged 41–60 and 61-80 (P < 0.001) (**Figure 6C**). Both p53 mutant and nonmutant UCEC patients exhibited significantly higher methylation levels of the CCND2 gene promoter compared to normal controls (P < 0.001), and notable differences were also observed between the two subtypes (P < 0.001, **Figure 6D**). These results show that the methylation levels of CCND2 gene promoter have a strong correlation with clinicopathological features in UCEC.

### 3.7 Relationship between the mRNA expression levels of CCND2 and clinicopathological features in UCEC

**Figure 7:**
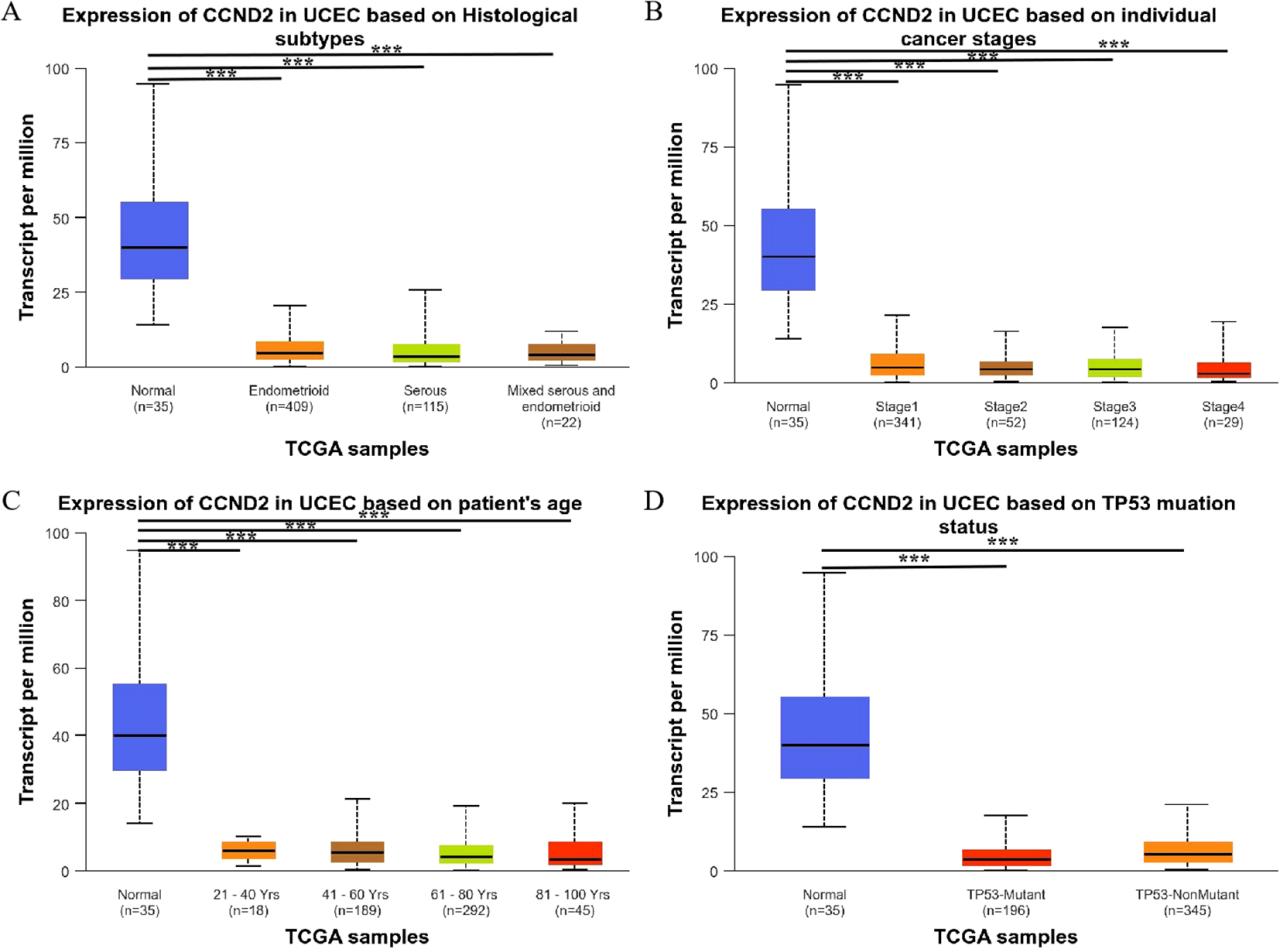
Relationship between the expression levels of CCND2 mRNA and clinicopathological features in UCEC (***P < 0.001).

Further analysis found that CCND2 mRNA levels were significantly reduced in different histological subtypes of UCEC compared to normal tissue, with a statistically significant difference (P < 0.001) (**Figure 7A**). Additionally, CCND2 mRNA levels were significantly lower in clinical stages 1–4 of UCEC (P < 0.001) (**Figure 7B**) and demonstrated a decline across different age groups of patients (P < 0.001) (**Figure 7C**). Both p53 mutant and nonmutant patients showed lower CCND2 mRNA expression than normal controls, with a significant difference (P < 0.001) (**Figure 7D**). These results show that the mRNA expression levels of CCND2 have a strong correlation with clinicopathological features in UCEC.

### 3.8 Analysis of the correlation between CCND2 mRNA expression and the expression of methylation regulatory factors

**Figure 8:**
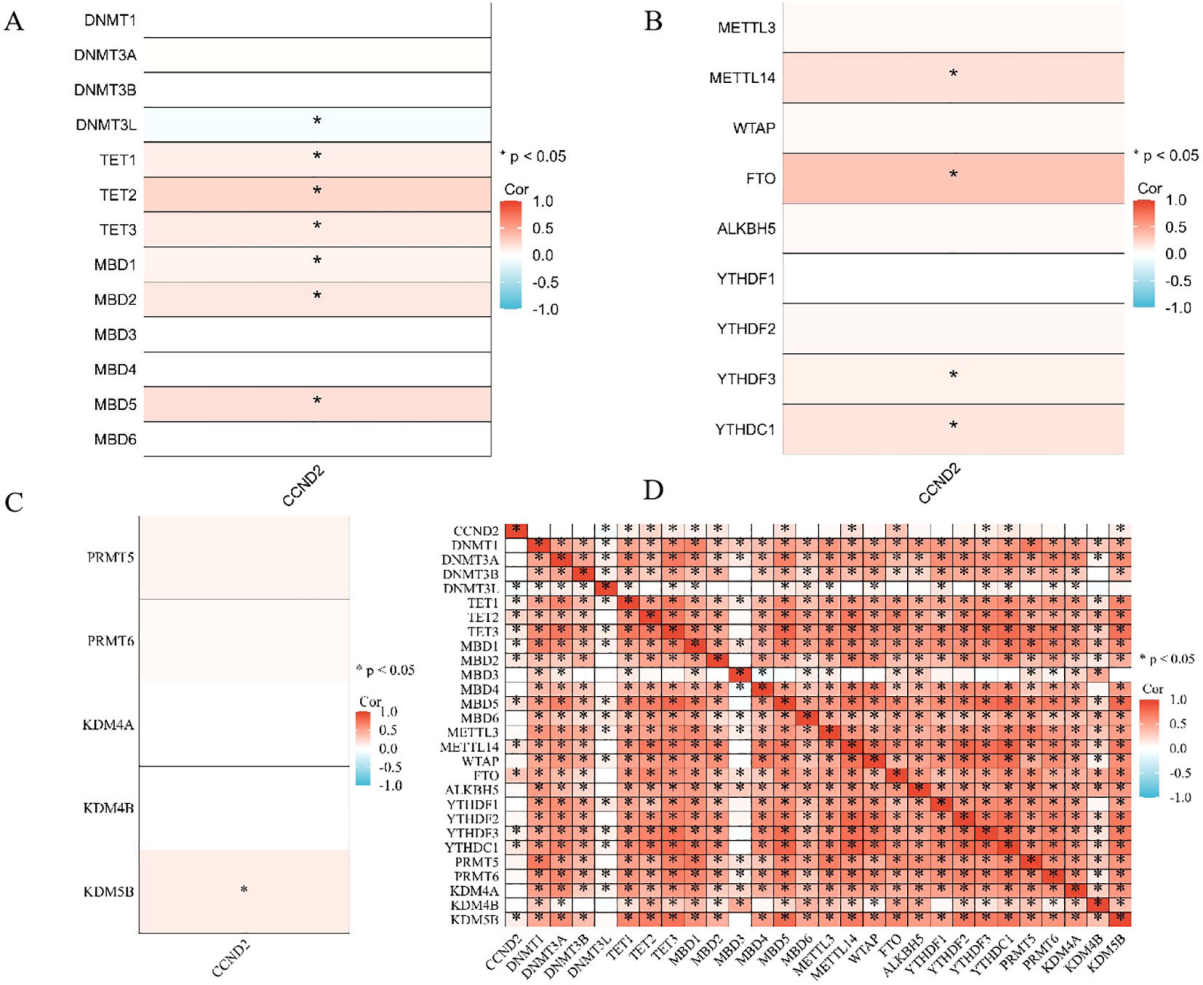
**(A)** Heatmap depicting the correlation between CCND2 expression and DNA methylation regulatory factors (*P < 0.05). **(B)** Heatmap showing the correlation between CCND2 expression and m6A methylation regulatory factors (*P < 0.05). **(C)** Heatmap showing the correlation between CCND2 expression and histone methylation regulatory factors (*P < 0.05). **(D)** Gene coexpression matrix (*P < 0.05).

A correlation analysis was performed using the R package on molecules related to CCND2 methylation, and the results were displayed in a heatmap. The analysis showed that CCND2 expression had a negative correlation with DNA methyltransferase 3-like protein (DNMT3L) (r = -0.0882, P < 0.05). In contrast, it exhibited a positive correlation with TET family demethylases (TET1, TET2, TET3) and methylation-binding proteins (MAD1, MAD2, MAD5) (r < 0.3, P < 0.05), as shown in **Figure 8A**. Furthermore, CCND2 expression positively correlated with m6A methyltransferase (METTL14), YTH binding protein 3 (YTHDF3), YTH domain-containing protein 1 (YTHDC1) (r < 0.3, P < 0.05), and KDM5B (r < 0.3, P < 0.05). Additionally, it showed a positive correlation with m6A demethylase (FTO) (0.3 ≤ r < 0.5, P < 0.01), as illustrated in **Figures 8B, C**. Gene coexpression correlation analysis showed that most of the proteins in the network had a strong positive correlation with each other (**Figure 8D**). Therefore, these CCND2 methylation-associated genes have a strong intertwined interaction and may be one of the reasons for the reduced expression of CCND2 protein in UCEC.

### 3.9 ROC curve analysis of the diagnostic efficacy and Cox survival analysis of CCND2 for UCEC

**Figure 9:**
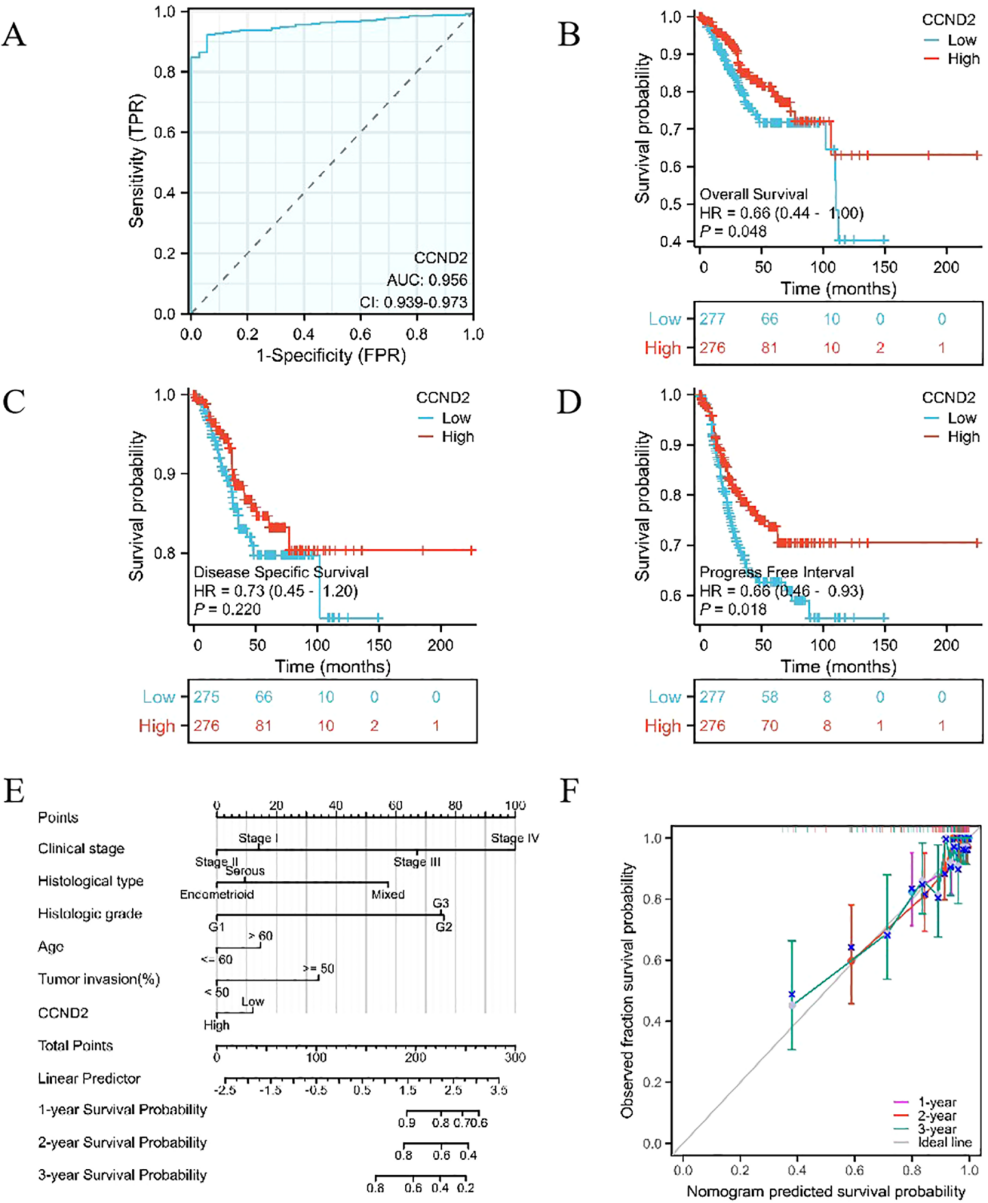
Providing an overview of various analyses involving CCND2. **(A)** ROC curve in UCEC. **(B)** KM survival curve (OS) for high and low CCND2 expression levels in UCEC. **(C)** KM survival curve (DSS) for high and low CCND2 expression levels in UCEC. **(D)** KM survival curve (PFI) for high and low CCND2 expression levels in UCEC. **(E)** Nomogram survival prediction chart for predicting the 1, 2 and 3 year overall survival rates. **(F)** Prognostic calibration curve.

RNA-seq data were downloaded and organized from the TCGA-UCEC project with the STAR workflow adopted. Subsequently, ROC analysis of the data was performed using the pROC package. The area under ROC curve (AUC) was a standard index for evaluating diagnostic tests. An AUC of 0.956 indicated that the expression of CCND2 had a good diagnostic effect, as shown in **Figure 9A**. Using survival package, univariate correlation analysis showed that 8 characteristics, stage III, IV, serous type, mixed type, G2 grade, G3 grade, tumor invasion (≥ 50%), and high CCND2 expression were significantly correlated with overall survival (OS). However, by multivariate analysis, the data showed that CCND2 expression (HR = 0.834, P = 0.450) was not an independent prognostic factor (**Table 3**). The proportional risk hypothesis was also evaluated and survival regression was performed. The results showed that high expression of CCND2 was correlated with extended OS and progression-free survival (PFI), and the significance level was P < 0.05, as shown in **Figures 9B–F**.

**Table 3:** Correlation between overall survival and multivariable characteristics in UCEC patients.

Characteristics	Total(N)	Univariate analysis		Multivariate analysis	
		Hazard ratio (95% CI)	P value	Hazard ratio (95% CI)	P value
Clinical stage	553				
Stage I	342	Reference		Reference	
Stage II	52	1.738 (0.833 - 3.626)	0.140	0.726 (0.249 - 2.122)	0.559
Stage III	130	3.084 (1.911 - 4.977)	<0.001***	3.006 (1.688 - 5.353)	< 0.001***
Stage IV	29	8.082 (4.497 - 14.524)	< 0.001***	6.388 (3.152 - 12.947)	< 0.001***
Histological type	553				
Endometrioid	411	Reference		Reference	
Serous	118	2.674 (1.744 - 4.100)	< 0.001***	1.412 (0.808 - 2.470)	0.226
Mixed	24	2.428 (1.040 - 5.672)	0.040*	3.517 (1.396 - 8.859)	0.008**
Histologic grade	542				
G1	99	Reference		Reference	
G2	121	7.111 (1.616 - 31.297)	0.009**	5.331 (1.196 - 23.775)	0.028*
G3	322	13.303 (3.262 - 54.242)	< 0.001***	5.282 (1.232 - 22.650)	0.025*
Tumor invasion(%)	475				
< 50	261	Reference		Reference	
>= 50	214	2.825 (1.752 - 4.554)	< 0.001***	2.075 (1.219 - 3.532)	0.007**
CCND2	553				
Low	277	Reference		Reference	
High	276	0.661 (0.438 - 0.996)	0.048*	0.834 (0.520 - 1.337)	0.450

*p < 0.05, **P < 0.01, ***P < 0.001.

## 4 Discussion

UCEC is a common malignant tumor in the female reproductive system, with an increasing incidence that significantly impacts women’s health and quality of life. Recent studies indicate that methylation changes are crucial in the occurrence and development of UCEC, as well as in assessing its prognosis (8). In-depth analyses of methylation patterns have revealed that specific patterns are closely linked to tumor aggressiveness, recurrence risk, and patient survival rates (9–11). These findings offer new insights into the molecular mechanisms of UCEC and pave the way for early diagnosis and personalized treatment approaches.

This study utilized a 935K chip for high-throughput methylation detection to analyze the methylation patterns in UCEC patients compared to normal control groups. As a result, 87,182 DMPs were identified. These DMPs spaned CpG islands, promoter regions, coding regions, and open chromatin regions across multiple chromosomes. They were primarily concentrated on chromosomes 1-18. Specifically, there were 22,483 differentially methylated sites in the promoter regions (Tss1500, Tss200, 3’UTR, 5’UTR, 1stExon), with 13,444 sites showing hypermethylation and 9,039 sites showing hypomethylation. DNA methylation is crucial for regulating gene expression. High methylation levels in promoter regions typically suppress transcription, whereas low levels promote gene expression. Abnormal methylation regulation facilitates cell proliferation, migration, and spread through various pathways. It also reduces the cells’ ability to repair DNA damage, weakens cell adhesion, and inhibits apoptosis and cell cycle arrest, which are vital for the occurrence and development of UCEC (8). Numerous studies have shown that various signaling pathways contribute to the occurrence, progression, and prognosis of UCEC. Research by Nout et al. highlights that the activation of oncogenic pathways like PI3K-Akt, Wnt/β-catenin, and P53 serves as a crucial prognostic factor for reduced disease-free survival (DFS) in UCEC patients (12). Moreover, CP41, a novel curcumin analog, triggers apoptosis in UCEC cells by activating the H3F3A/proteasome-MAPK signaling pathway and boosting oxidative stress (13). Additionally, the glucagon-like peptide-1 receptor (GLP1R) hinders UCEC progression by activating the cAMP/PKA pathway (14). The KEGG pathway enrichment analysis from this study revealed that these DMPs primarily participated in the PI3K-Akt, MAPK, and cAMP signaling pathways. This suggests that these signaling pathways and their key factors in cell cycle regulation may play a role in the initiation and advancement of UCEC through DNA methylation modifications.

In mammalian cells, D-type cyclins (CCND1, CCND2, CCND3) are encoded by different genes on three chromosomes. CCND2 is unique among the three cyclins, as it is located on chromosome 12p13. It consists of five exons that encode the CCND2 protein, which plays a crucial role in the PI3K-Akt-mTOR pathway. It binds with cyclin-dependent kinases 4/6 (CDK4/6) to activate the formation of a complex. This process causes the Rb protein to be phosphorylated and inactivated, thereby releasing the transcription factor E2F and promoting the transition of cells from the G1 phase to the DNA synthesis phase (15). In addition to regulating the cell cycle, CCND2 is also closely related to cell differentiation and tumor transformation (16). Abnormal expression of CCND2 is strongly associated with the occurrence, development, and prognosis of various tumors. However, studies yield inconsistent results regarding its expression levels. While the accumulation of CCND2 is often linked to the onset of certain diseases, many cancers, particularly breast and lung cancer, are associated with reduced expression due to excessive methylation of CCND2 (17). It has been reported that hypermethylation of the CCND2 promoter can be detected in the early stages of breast cancer and is associated with its expression silencing (18). Using demethylating agents can increase CCND2 expression in breast cancer samples and inhibit cancer cell growth by inducing cell cycle arrest (17). It’s still unclear why CCND2 is absent in cancer, yet cell proliferation related to CCND2 is observed. This may be due to a compensatory effect leading to the upregulation of another cyclin or may be related to different stages or subtypes of cancer. In gastric cancer, some studies suggest that high methylation of CCND2 may promote cellular proliferation (19). Conversely, other research indicates that low methylation of CCND2 is associated with increased CCND2 expression in advanced stages of gastric cancer (20). These findings illustrate the complexity and importance of CCND2 in cell cycle regulation, tumorigenesis and prognosis assessment. Although various genes such as ADCYAP1, HAND2, MME, and RASSF1A have been reported to exhibit abnormal methylation and tumor development in UCEC (21–23), the role of CCND2 gene methylation in UCEC has not been reported. Therefore, the primary focus of our research is the methylation and expression levels of CCND2 in UCEC and their clinical significance.

This study found that methylation levels of several CCND2 CpG islands were higher in UCEC tissues, as detected by 935K chip analysis. Additionally, IHC detection revealed a significant reduction in CCND2 protein expression. Subsequent bioinformatics analysis demonstrated a significant increase in the methylation level of CCND2 in UCEC compared to normal controls, consistent with the chip results. Conversely, bioinformatics analysis indicated that its mRNA expression was significantly reduced. Furthermore, the methylation level of the CCND2 gene promoter region exhibited a negative correlation with its mRNA expression. Further investigation into the relationship between CCND2 gene methylation, mRNA expression, and clinicopathological features provided valuable insights into disease mechanisms. The findings show that UCEC has significantly abnormal DNA methylation patterns and expression profiles. The hypermethylation of the CCND2 promoter region may reduce the CCND2 expression in UCEC. However, it is unclear how high levels of methylation in the CCND2 promoter lead to gene silencing. Moreover, the potential impacts of other epigenetic modifications, stress, hormonal changes, and physiological cycles on CCND2 expression need further exploration. This study analyzed the correlation between CCND2 expression and methylation regulatory factors using R packages. The results indicated that reduced CCND2 expression in UCEC patients may be linked to increased DNMT3L expression and decreased TET family demethylase expression. Additionally, m6A modification and histone methylation may also influence CCND2 expression.

DNA methylation markers show significant abnormal methylation changes in tumor cells. These changes occur during cancer initiation and progression and provide critical insights for studying tumor biology. Certain DNA methylation anomalies typically emerge in the early stages of tumor formation. This offers significant opportunities for early cancer diagnosis and monitoring treatment responses. Additionally, it helps assess disease progression risks through the detection of methylation markers strongly associated with tumorigenesis (24, 25). This study thoroughly evaluated the high methylation status of the CCND2 promoter region in UCEC tissues and the significantly reduced mRNA expression level, which was considered an early event in tumor occurrence and may serve as a potential biomarker for early diagnosis of UCEC. ROC analysis and survival analysis results showed that high expression of CCND2 was associated with longer overall survival and progression-free survival, further emphasizing its importance in tumor prognosis assessment.

## 5 Conclusions

In summary, the study shows that UCEC has significantly abnormal DNA methylation patterns and expression profiles through methylation chip and bioinformatic analysis. The hypermethylation of the CCND2 promoter region in UCEC tissues is negatively correlated with low mRNA expression, indicating that high methylation may suppress CCND2 gene expression and participate in tumor occurrence and development. Additionally, hypermethylation and reduced expression of CCND2 are recognized as early events in tumorigenesis, associated with UCEC survival and prognosis. Hence, CCND2 shows promise as a potential biomarker for diagnosing and prognosticating UCEC.

## Data Availability

The datasets presented in this study can be found in online repositories. The names of the repository/repositories and accession number(s) can be found in the article/**Supplementary Material**.
