# Supplementary material for: Cyclin D2 – a potential biomarker in uterine corpus endometrial carcinoma through methylation chip and bioinformatic analysis
Source: Front Oncol. 2025 Jul 3;15:1569782. doi: 10.3389/fonc.2025.1569782 (PMC12267004; doi:10.3389/fonc.2025.1569782)
Supplement: Supplementary file 1 [file DataSheet1.docx]

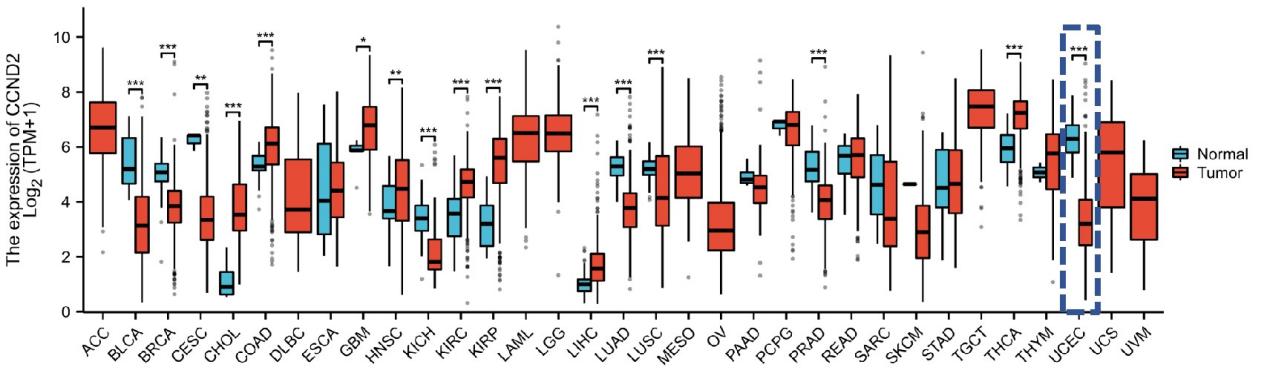


FIGURE S1

Increased or decreased CCND2 expression in datasets of different cancers compared with normal tissues in TCGA database.
